# Testing the HAPA model for predicting daily physical activity of women survivors of breast cancer

**DOI:** 10.1177/13591053251347143

**Published:** 2025-07-20

**Authors:** Margarida Sequeira, Cícero Pereira, Maria-João Alvarez

**Affiliations:** 1 Universidade de Lisboa, Portugal; 2 Instituto Politécnico de Setúbal, Portugal; 3 Instituto de Ciências Sociais da Universidade de Lisboa, Portugal

**Keywords:** HAPA, N-of-1 design, oncology, physical activity, survivors of breast cancer

## Abstract

The power of the Health Action Process Approach (HAPA) for understanding interindividual health outcomes is well-documented. However, its applicability in explaining intraindividual evolution of constructs over time remains under-researched. This study addresses this gap by conducting nine longitudinal N-of-1 studies to estimate how the HAPA constructs predict physical activity (PA) at the intraindividual level in breast cancer survivors over-time. PA actual behaviors (*N* = 338) of nine women survivors of breast cancer were observed over 6-weeks, and associations between HAPA constructs and PA were analyzed longitudinally. Time-series regression analyses revealed that self-efficacy and social-support significantly predicted participants’ intention to engage PA. Recovery self-efficacy emerged as predictor of PA in three studies, while planning was predictor in one. Future interventions targeting PA after breast cancer should consider individual and daily variations, and should focus self-efficacy, social-support, and planning. This highlights the importance of tailoring interventions to promote PA to specific and individual needs.

## Introduction

There is robust evidence of the systemic benefits of physical activity (PA) for patients with breast cancer, leading to increased aerobic fitness, improved quality of life, better activity tolerance, and reductions in fatigue, depressive symptoms, and therapeutic toxicity in survivors of breast cancer (Furmaniak et al., 2016; Lahart et al., 2018; Patel et al., 2019). However, breast cancer diagnoses and treatments often contribute to a decline in PA (Bluethmann et al., 2015; Harris et al., 2012; Spark et al., 2013). This presents a significant challenge to the scientific community in developing effective strategies to address this propensity for lower levels of PA.

To promote behavior change toward a more active lifestyle among survivors of breast cancer it is critical to identify the specific components of interventions that facilitate such change (Bluethmann et al., 2015). Behavior change theories have been proposed as valuable frameworks for improving interventions by linking relevant causal factors to appropriate behavior change techniques (Buffart et al., 2014; Michie et al., 2013). One of the models tested and demonstrated for health behavior change is the Health Action Process Approach (HAPA; Schwarzer, 2008), which integrates both motivational and volitional constructs, as it does not regard intention alone as a sufficient tool for self-regulation. It has been shown to be of good predictive validity across a variety of different health-related behaviors, including health-enhancing behaviors such as PA (Schwarzer and Luszczynska, 2015).

The HAPA considers that during the motivation phase, individuals develop an intention to act, with *risk awareness* (awareness of a health threat), *outcome expectancies* (the pros and cons of a particular behavior), and *action self-efficacy* (an optimistic belief in one’s own personal ability to initiate such changes) identified as predictors of intentions. Subsequently, intentions must be transformed into detailed *action plans* that specify when, where, and how the desired behaviors will be executed. Planning also includes *coping planning* which requires identifying obstacles that might jeopardize the intended behavior and developing strategies to overcome them. *Planning* and *action control* (self-regulatory skills and strategies) contribute to translating intentions into actions. Individuals face phase-specific demands, and therefore different phase-specific self-efficacy beliefs are distinguished to cope with those challenges (Schwarzer, 2008). *Maintenance self-efficacy* reflects optimistic beliefs in one’s ability to maintain a health behavior in the face of barriers and obstacles. In addition, *recovery self-efficacy* refers to an individual’s confidence in his or her ability to return to the intended behavior after setbacks, especially in complex situations. The HAPA model provides an open framework that allows for the inclusion of other variables such *social support*, which has been shown to influence intentions and behaviors, particularly in the context of PA, including in survivors of breast cancer (Fernández et al., 2014; Parschau et al., 2014).

Recent theories and models of behavior change, including the HAPA, have emphasized the importance of shifting the focus of analysis from interindividual studies to longitudinal time series observations within participants (Quinn et al., 2013). This approach provides a more comprehensive understanding of how individual-specific factors that influence behavioral outcomes are critical for developing more reliable interventions (Kwasnicka and Naughton, 2020).

N-of-1 designs have been recognized as a critical tool for testing theories because they involve the systematic repeated measurement of specific variables within an individual over time (Craig et al., 2008), making it possible to identify the relationship between predictors and individual-level behavior over a duration (Kwasnicka et al., 2019). N-of-1 designs allow for an examination of whether the pattern of relationships among variables in the model is similar or different for each participant over time through repeated and individualized analysis.

A previous time-series multilevel study by Sequeira et al. (2023) investigated the interindividual longitudinal evolution of HAPA model constructs and their relationship to PA in breast cancer survivors. The results provided compelling evidence for the predictive power of the HAPA constructs for PA intentions and behavior in this population over time. In particular, *self-efficacy* and *social support* consistently predicted PA intentions, while *planning* and *recovery self-efficacy* were particularly effective in predicting actual PA engagement.

While Sequeira et al. (2023) focused exclusively on interindividual time-varying HAPA constructs as predictors of participants’ PA, they did not examine how the model worked to promote PA within each specific participant over time. Therefore, it remains unclear whether the patterns identified at the group-based interindividual level are equally effective for each individual survivor. Addressing this gap could provide groundbreaking empirical insights and extend the heuristic power of the HAPA model to understand health-related behaviors at the intraindividual level.

The present study aims to address this concern for the first time through a secondary analysis of the Sequeira et al. (2023) dataset. Instead of an aggregate interindividual analysis, nine studies based on an N-of-1 time-series longitudinal design were conducted, which allowed us to derive robust estimates of the relationships between the main HAPA constructs in predicting daily PA at the intraindividual level among breast cancer survivors over time.

## Methods

### Design and participants

Nine studies using an N-of-1 design were conducted, with each study comprising *N* = 42, which corresponds to the number of days on which the HAPA variables and behaviors were observed, yielding 338 valid data points for the actual PA behavior and HAPA constructs. Although the sample size of observed behaviors in each study was relatively small, it was sufficient to detect an effect size of *r* = 0.43 or greater at *p* < 0.05, as estimated using WebPower (Zhang and Yuan, 2018).

The study spanned from April to July 2019, with daily and weekly observations conducted over a 6-week period. Eligibility criteria for participation included adult women aged 18–64 years, whose breast cancer treatment had been completed, and with literacy and cognitive skills to provide informed consent.

### Procedures

The participants were recruited through convenience sampling from cancer patient associations and snowball sampling. A face-to-face meeting was conducted to ensure informed consent and to clarify the study objectives, potential benefits, and rights of participants. During this individual meeting, participants completed an online questionnaire including sociodemographic and clinical data previously identified as relevant to regular PA in survivors of breast cancer (Hefferon et al., 2013; Kampshoff et al., 2014). To ensure confidentiality, each participant was assigned a unique code.

In the individual meeting, data collection process was explained, and participants were given a Xiaomi Mi Band 2^®^ pedometer. Previous research has demonstrated the validity and reliability of this device for measuring step count during PA (Martínez-Martínez et al., 2020; Pino-Ortega et al., 2021). Participants were instructed to wear the pedometer every day to record their daily step count, during the 6-week length of the study. Weekly telephone contact was established to clarify doubts and address any concerns.

An Ecological Momentary Assessment (EMA) approach was used to collect daily and weekly data over a 6-week period (Marszalek et al., 2014). Participants could choose email or text messaging for data collection, depending on personal preference.

To ensure the reliability and suitability of the questionnaires and procedures, a pilot study was conducted with five people. Their feedback was used to refine and adjust the questionnaires and daily and weekly assessment procedures. The study received ethical approval from the Ethical Committee of the Faculty of Psychology of the University of Lisbon.

### Measures

Three sets of measurements were performed using EMA. The first set included daily assessment of HAPA constructs over 42 days *(behavior intention, action self-efficacy, action planning, coping planning, maintenance self-efficacy, action control* and *social support*). The second set included weekly assessment of the HAPA constructs over a 6-week period (*risk awareness, outcome expectancies and recovery self-efficacy*). The temporal observation of the variables was based on theoretical and empirical information, and the weekly measured were considered not subject to daily fluctuation as evidenced in previous studies (Loehr et al., 2014). The third set included the assessment of daily PA, thought step counts.

The HAPA constructs were measured using a response scale ranging from 1 (strongly disagree) to 6 (strongly agree). The items used for measurement were adopted from previous PA studies (Bierbauer et al., 2017; Scholz et al., 2009), and all items were considered: *behavioral intentions*, with the item: “I intend to practice physical activity tomorrow and contribute to the weekly 150 minutes”; *risk awareness*, was measured by the item: “I know I may have new health problems related to breast cancer”; *outcome expectancies*, with the item: “I feel that by doing at least 150 minutes of physical activity a week I can improve breast cancer related problems”; for *action self-efficacy* the item was: “I’m confident that tomorrow I will be able to practice physical activity”; for *maintenance self-efficacy*: “I’m confident that tomorrow I will be able to practice physical activity, even if difficulties arise”; for *social support*: “I have someone to support me in carrying out the physical activity scheduled for tomorrow”; for *planning* the items were: “I’ve already planned when, where, and how to be able to practice physical activity tomorrow” (action planning) and “I have plans to overcome the barriers that may arise tomorrow to prevent me from practicing physical activity” (coping planning); for *recovery self-efficacy*: “Once I start practicing regular physical activity, I will be able to maintain this practice, even if I stop for a while”; *action control* was assessed by two items, one for the awareness of one’s own standards: “Today I was aware of the goal I set myself to practice physical activity and contribute to the 150 minutes per week”; the other for self-regulatory effort: “Today I made an effort to meet my intention to practice physical activity, in order to contribute to the weekly 150 minutes”. Unlike the other constructs, *action control* was asked about in reference to the PA performed on that current day (see Supplemental Appendix 1).

Some theoretical aspects were explored regarding a specific issue: whether respondents could discern small differences between the items measuring strongly related HAPA concepts (Godinho et al., 2014; Hagger and Luszczynska, 2014; Schwarzer and Luszczynska, 2015). To investigate this, an independent sample of 11 women participated in a pilot study. They were tasked with grouping items based on similarities and differences (see the Supplemental Material for detailed explanations of this pilot study—Supplemental Appendix 2). Participants organized the daily constructs into the following groups: *behavioral intentions; self-efficacy* (combining action self-efficacy and maintenance self-efficacy); *planning* (combining action planning and coping planning); *action control* (combining awareness of personal standards and self-regulatory effort); and *social support*. These grouped constructs were ultimately used in the analysis. The HAPA constructs considered in this analysis are those identified in a previous study (Sequeira et al., 2023) as reliable predictors of PA intentions, namely *self-efficacy* and *social support*, and of PA behaviors, including *planning* and *recovery self-efficacy*, among breast cancer survivors.

The daily PA was measured using daily step counting by the pedometer. When PA or exercise was performed without a pedometer (e.g. swimming, kickboxing), the data were converted to “step equivalents” by using the specific metabolic equivalent of the task (MET) of the activity (Ainsworth et al., 2011; Miller et al., 2006) and combined with raw step counts to obtain an overall estimate of steps per day.

### Data analysis strategy

Nine single-case time series regression analyses were conducted for each dependent variable: PA intention and PA behavior, delving deeper the data by analyzing how specific constructs—identified in the previous longitudinal study as predictors of PA intentions (*self-efficacy* and *social support*) and PA behaviors (*recovery self-efficacy* and *planning*)—influence PA on an individual level among women who have survived breast cancer. Each analysis incorporated the estimation of the autoregression effect and the linear time trend to account for potential cumulative carry-over effects associated with each outcome variable. The pattern of results was synthesized by calculating the statistical effects derived from the set of nine studies.

## Results

### Intention to perform PA

**Figure 1.**
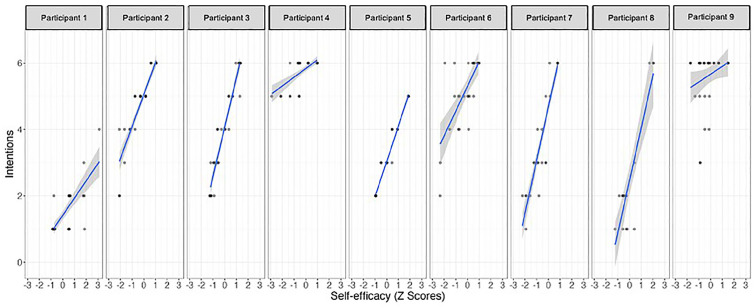
Relationship between self-efficacy and behavioral intentions in each participant.

**Figure 2.**
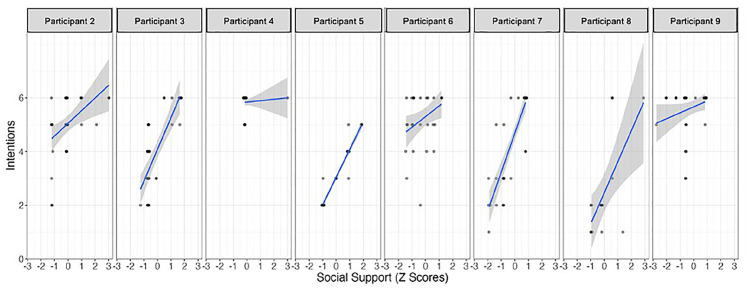
Relationship between social support and behavioral intentions in each participant.

The results of the single-case time series regression analyses indicate that *self-efficacy* and *social support* significantly predicted participants’ intention to engage in PA, but not for all participants (Table 1 and Figures 1 and 2). Notably, *self-efficacy* showed to be a more effective predictor, with seven out of the nine participants exhibiting a positive relationship between their self-reported belief in potential involvement in PA and their intention to engage in PA on the same day. Moreover, Participant 2 displayed a reliable autoregressive effect, whereby her intention to engage in PA on a given day influenced her intention on the subsequent day. The impact of *social support* on intention to engage in PA was less pronounced, demonstrating a significant positive association only for Participants 5 and 7. These participants reported a greater intention to engage in PA on days when they perceived higher levels of social support. The estimated effect of time was not a significant predictor of intention PA.

**Table 1.** Unstandardized regression parameter estimated in the single case analysis for PA intentions.

Participant	Self-efficacy		Social Support		Time		Autocorrelation Lag_1	
	*b*	SE	*b*	SE	*b*	SE	*b*	SE
1	0.841	0.244			−0.004	0.007	0.141	0.116
2	0.833***	0.060	−0.040	0.071	−0.006	0.005	0.117*	0.056
3	0.962***	0.072	−0.041	0.070	−0.004	0.007	−0.041	0.049
4	0.412***	0.072	−0.022	0.061	−0.001	0.004	−0.006	0.130
5	0.653***	0.084	0.334***	0.085	0.000	0.003	0.031	0.036
6	0.750***	0.137	−0.154	0.088	0.024	0.013	0.061	0.121
7	0.820***	0.082	0.260**	0.068	−0.001	0.006	−0.073	0.042
8	1.045***	0.200	0.134	0.226	0.040	0.044	0.148	0.097
9	0.134	0.129	0.098	0.111	0.007	0.011	−0.035	0.165

* *p* < 0.05. **p < 0.01. ***p < 0.001.

### Actual PA

**Figure 3.**
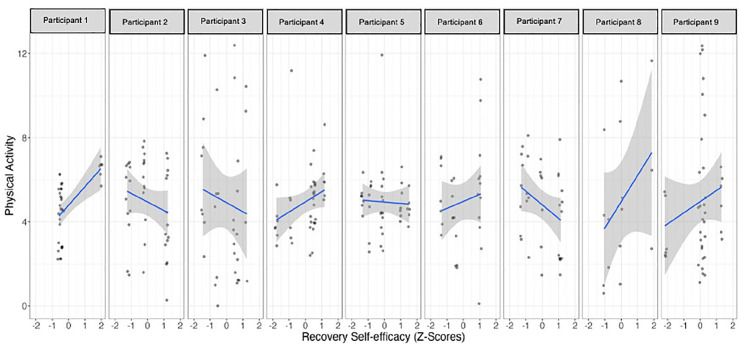
Relationship between recovery self-efficacy and physical activities in each participant.

**Figure 4.**
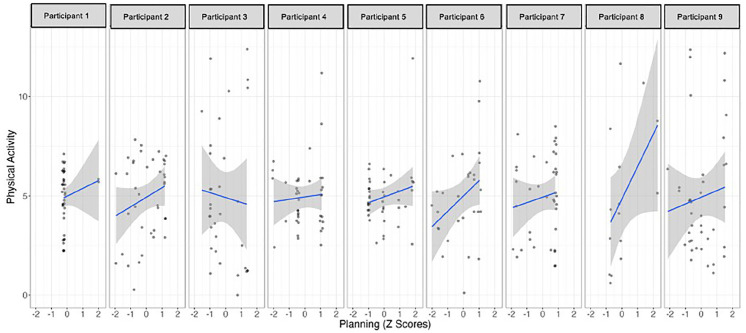
Relationship between planning and physical activities in each participant.

The single-case time series regression analyses showed that *recovery self-efficacy* positively predicted PA for three out of the nine participants (Table 2 and Figures 3 and 4). Participants 1 and 4 demonstrated a significant association between their confidence in the ability to return to regular behavior and their engagement in PA on a given day, with higher levels of self-efficacy corresponding to increased PA. Conversely, Participant 7 displayed a unique negative effect, indicating that higher belief in recovery self-efficacy was associated with decreased engagement in PA. Notably, Participant 7 exhibited a reliable negative autoregressive effect, suggesting a carry-over effect where increased PA on one day led to decreased PA on the subsequent day. *Planning* emerged as a significant predictor of PA for participant 5, with higher reported planning activities associated with increased engagement in PA. The duration of the study was found to be a relevant predictor for participants 4 and 9, with their engagement in PA increasing over the course of the study.

**Table 2.** Unstandardized regression parameter estimated in the single case analysis for PA behavior.

Participant	Recovery self-efficacy		Planning		Time		Autocorrelation Lag_1	
	*b*	SE	*b*	SE	*b*	SE	*b*	SE
1	1.692*	0.671	0.426	0.870	0.038	0.026	0.248	0.186
2	−0.675	0.498	0.426	0.289	0.018	0.029	−0.048	0.179
3	−3.820	2.639	−0.019	0.401	0.135	0.119	−0.366	0.187
4	1.464*	0.568	−0.071	0.353	0.077**	0.025	0.055	0.137
5	−0.011	0.369	1.019**	0.312	−0.034	0.029	0.008	0.157
6	−0.639	0.982	0.207	0.257	0.048	0.042	0.151	0.166
7	−1.178*	0.476	0.163	0.195	0.044	0.035	−0.378*	0.183
8	3.497	2.975	0.822	0.637	−0.302	0.283	−0.238	0.273
9	2.930	2.400	0.411	0.417	0.127*	0.053	0.020	0.167

* *p* < 0.05; **p < 0.01.

**Figure 5.**
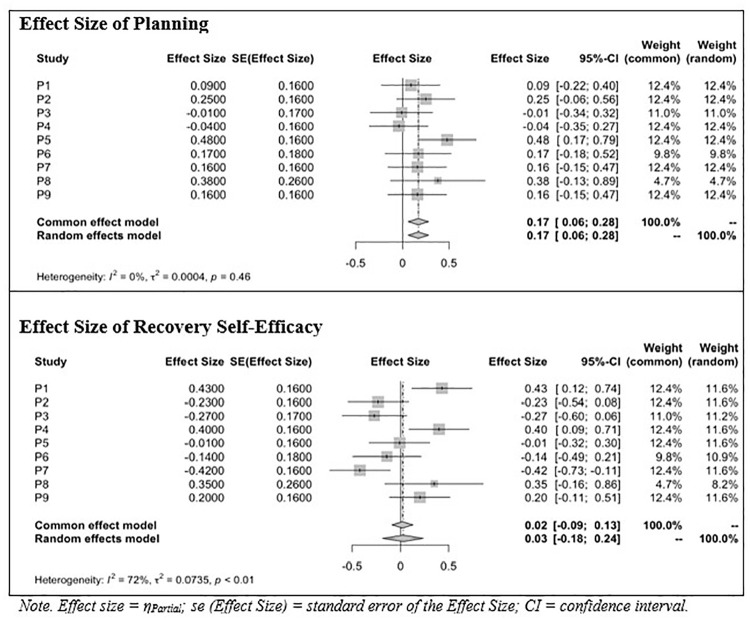
Forest plot of meta-analytic effect size of planning and recovery self-efficacy on PA.

Figure 5 shows the meta-analytic results for *planning* (upper section) and *recovery self-efficacy* (lower section). Planning showed a consistent positive effect across the studies, with a meta-analytic effect size of *r* = 0.17 and no significant variability between studies. In contrast, recovery self-efficacy showed a weaker overall effect (*r* = 0.02), but showed significant variability between studies. This variability suggests the potential influence of moderating factors affecting the relationship between individuals’ recovery self-efficacy and physical activity (PA).

## Discussion

The constructs that were found to be predictors of PA intentions (*self-efficacy* and *social support*) and PA behaviors (*recovery self-efficacy* and *planning*) in a previous longitudinal study conducted with women who had survived breast cancer (Sequeira et al., 2023) were included in the current study at a deeper intraindividual level of data analysis by estimating these effects on PA in each of the participants. Time effects in the variation of the constructs were also examined, aiming to control whether the estimated effect of predictors on the dependent variables was due to a mere time effect (Bierbauer et al., 2017).

Daily fluctuations in self-efficacy were found to play a consistent role in the intentions to become more active, for seven of the nine participants, which is in line with the results obtained in a previous study (Sequeira et al., 2023) and consistent with the HAPA model theoretical description (Schwarzer, 2008).

Social support significantly influenced PA intentions but only for two of the nine participants and it was observed that the strength and direction of the relationship between social support and PA intentions varied widely. This contrasts with previous findings, in which social support was identified as a key factor for PA intentions (Parschau et al., 2014), and in this specific sample after 12 days of a 42-day observation period (Sequeira et al., 2023), demonstrating a positive interaction with time. We put forward at the possibility that, at this intraindividual level of data analysis, the 42-day observation period was not sufficient for the effect of social support to fully emerge, potentially due to low statistical power. Additionally, social support has been shown to play a more consistent role in actual PA behavior, rather than in the formation of intentions, but with different results depending on the support provider being a family member or a friend (Lindsay Smith et al., 2017; Rackow et al., 2014). In the same line, a systematic review where different sources of social support influence in PA behavior were investigated (family, friends, both, peers or significant others) identified that this relationship varied widely, ranging from small to large and from negative to positive (Scarapicchia et al., 2017). Furthermore, research shows that when self-efficacy is high, the level of social support does not significantly impact self-regulation of behavior (Fernández et al., 2014; Maher and Conroy, 2016), and all the women of our study demonstrated strong self-efficacy, both motivational and volitional. A closer intraindividual analysis revealed that the two participants for whom social support was crucial in setting PA intentions had longer post-diagnosis periods (13 and 18 years). This may suggest that social support becomes more important for PA intentions when PA is no longer viewed as essential for survival, but rather as a matter of personal convenience. Despite previous findings on the role of social support for this group of women (Sequeira et al., 2023), research suggest that social support is not always critical for fostering PA intentions and may not be universally beneficial for every breast cancer survivor PA behavior (Lindsay Smith et al., 2017; McDonough et al., 2021; Scarapicchia et al., 2017), suggesting an individualized approach to this construct in general and type of support and provider in particular.

Participant 2 was the only one for whom the intention to engage PA on a given day was reliably associated with her intention on the subsequent day, in a negative way. This may be related to her job that demands physical efforts, standing and walking around, which may cause extra fatigue at the end of the day, preventing her intentions to be more physically active on the following day. Actually, fatigue has long been recognized as a significant barrier to regular PA among cancer survivors (Hefferon et al., 2013).

When the PA behavior was under analysis, recovery self-efficacy positively predicted PA, for participants 1 and 4. Indeed, previous analysis identified that recovery self-efficacy predicted PA differently over time, so that the stronger the recovery self-efficacy, the more kilometers the participants walked (Sequeira et al., 2023). In this deeper intraindividual analysis these two participants have the common characteristics of being not married, with very low (or no) social support declared throughout the study. As mentioned, social support has been identified in the literature as important in the volition phase for PA behaviors when self-efficacy is lower, helping to cope with the stressful demands which come after an intention is set and acting as a buffer (Parschau et al., 2014). It may be that the low perceived social support, engaged their self-efficacy, and mostly recovery self-efficacy throughout the study, predicting more PA for these women with 4 and 6 years after breast cancer diagnosis and who stated to be active before breast cancer, but not after.

Conversely, for participant 7, higher beliefs in recovery self-efficacy were associated with decreased engagement in PA, with social support playing the significantly important effect on her intentions to become active. Notably, this participant exhibited a reliable negative autoregressive effect, suggesting a carry-over effect where increased PA on one day led to decreased PA on the subsequent day. This may be related with the fact that this woman, 18 years after breast cancer diagnosis, has her PA habits well established, with good perceived social support, and may feel that she can return to PA whenever she wants, even after a relapse (Parschau et al., 2014; Schwarzer and Luszczynska, 2015).

Considering the role of planning in the prediction of PA behavior, it only emerged as significant for participant 5, with higher reported planning activities associated with increased engagement in PA. This contrasts with the group analysis of the data, where planning was found to be a significant factor in achieving higher levels of physical activity (Sequeira et al., 2023) and with other N-of-1 studies where planning was identified as important to the establishment of regular PA (Fernández et al., 2014; Parschau et al., 2014). It underscores the importance of deeper individual analyses to better understand the PA determinants for specific women (Kwasnicka and Naughton, 2020). This 54-year-old woman does her PA with her husband, so social support might import for her intention to become active (as previously mentioned), and planning was fundamental for her consistent PA practice. Despite this positive result for participant 5, the remaining individual results were not to be expected, because previous data for the entire sample identified that participants walked more kilometers on the days they did more planning of their activities. Additionally, both action and coping planning have been identified as important for increasing PA in survivors of breast cancer (Paxton, 2016). The model of planning can sometimes be limited when individuals have great confidence in their ability to put the behavior into practice (Maher and Conroy, 2016), and this is precisely what was found in the women in the sample, with high levels of self-efficacy.

Participants 4 and 9 increased their PA levels over the course of the study, suggesting that time may play a crucial role for women who were physically active before their cancer diagnosis but feel they have not yet fully regained their previous activity levels. This finding aligns with known determinants of PA among breast cancer survivors, such as pre-diagnosis PA habits (Kampshoff et al., 2014) and highlights the importance of allowing women the time to recover their well-being before gradually resuming regular PA (Costanzo et al., 2011).

### Study limitations

The study is subject to limitations. The close interaction between the first author and the participants may have influenced the participants’ perceptions of social support, with the researcher potentially substituting their natural sources of support, thus limiting the role of social support as a significant predictor in this analysis. Additionally, the limited number of daily and weekly observations might explain why behavioral predictors were significant for only four participants. Furthermore, the study was conducted between April and July, during favorable weather conditions, which may have influenced the identification of some variables as predictors of physical activity.

## Conclusions

The pattern of results represents an important improvement in our findings on the suitability of the HAPA model for predicting and promoting consistent PA at the individual level. Particularly, to our knowledge the applicability of the model to understanding the role of the model variables in particular cases in time series longitudinal design was presented. The model showed to be explanatory of individual behavior, in this case, of female breast cancer survivors, both in terms of predictors of intention and predictors of behavior. The study contributes to understanding the nuances of the model due to the design and population used.

Furthermore, it impacts on the theoretically understanding of PA promotion, the specificity of breast cancer survivors, and highlights some contribution to adding nuances to the HAPA model. Particularly, it emphasizes the importance of taking daily and individual variations into account when designing future interventions. Considering that many of the constructs assessed vary significantly on a daily basis, interventions can benefit from this sensitivity to frequent variations in order to adapt them to the needs of individuals, making the HAPA a useful model for customized interventions with breast cancer survivors. Interventions targeting social support, planning, and recovery self-efficacy may prove being differently effective in promoting PA behaviors in individual women breast cancer survivors. This differentiated approach is critical to developing interventions that are not only effective but also tailored to the unique needs of this population.

## Supplemental Material

sj-docx-1-hpq-10.1177_13591053251347143 – Supplemental material for Testing the HAPA model for predicting daily physical activity of women survivors of breast cancer Supplemental material, sj-docx-1-hpq-10.1177_13591053251347143 for Testing the HAPA model for predicting daily physical activity of women survivors of breast cancer by Margarida Sequeira, Cícero Pereira and Maria-João Alvarez in Journal of Health Psychology

sj-docx-2-hpq-10.1177_13591053251347143 – Supplemental material for Testing the HAPA model for predicting daily physical activity of women survivors of breast cancer Supplemental material, sj-docx-2-hpq-10.1177_13591053251347143 for Testing the HAPA model for predicting daily physical activity of women survivors of breast cancer by Margarida Sequeira, Cícero Pereira and Maria-João Alvarez in Journal of Health Psychology
