# Supplementary material for: Testing the HAPA model for predicting daily physical activity of women survivors of breast cancer
Source: J Health Psychol. 2025 Jul 20;31(3):1104–19. doi: 10.1177/13591053251347143 (PMC12949744; doi:10.1177/13591053251347143)
Supplement: sj-docx-1-hpq-10.1177_13591053251347143 – Supplemental material for Testing the HAPA model for predicting daily physical activity of women survivors of breast cancer [file sj-docx-1-hpq-10.1177_13591053251347143.docx]

| **Supplemental File 1 – Questionnaires and items in English and Portuguese**  **1.1 – Daily questionnaire in Portuguese** | |
| --- | --- |
| Este questionário pretende conhecer melhor a forma como se sente face à prática de atividade física diária e a quantidade de atividade física que pratica.  Ocupa cerca de 2 minutos a preencher e **deve ser preenchido ao fim do dia.**  Para as primeiras 8 questões, utilize a escala de 6 pontos, selecionando a opção que mais se adequa a si de acordo com os seguintes significados: 1 "Discordo totalmente"; 2 "Discordo bastante"; 3 "Discordo um pouco"; 4 "Concordo um pouco"; 5 "Concordo bastante"; 6 "Concordo totalmente"  A partir da pergunta 9 preencha os espaços de resposta de acordo com o que foi a Atividade Física realizada hoje. **Muito obrigada pela colaboração.** | |
| **Constructo** | **Questão** |
|  | De modo a ir ao encontro das recomendações da Organização Mundial de Saúde de fazer pelo menos 150 minutos de atividade física semanal: |
| **Intenção** | 1. Tenho intenção de praticar atividade física amanhã e contribuir para os 150 minutos semanais   1 2 3 4 5 6  Discordo totalmente O O O O O O Concordo totalmente |
| **Auto-eficácia de ação** | 1. Estou confiante que amanhã serei capaz de praticar atividade física   1 2 3 4 5 6  Discordo totalmente O O O O O O Concordo totalmente |
| **Planeamento de ação (action planning)** | 1. Já planeei quando, onde e como conseguir praticar atividade física amanhã   1 2 3 4 5 6  Discordo totalmente O O O O O O Concordo totalmente |
| **Identificação de estratégias (planeamento de coping)** | 1. Tenho planos para ultrapassar as barreiras que amanhã possam surgir e que me impeçam de praticar atividade física   1 2 3 4 5 6  Discordo totalmente O O O O O O Concordo totalmente |
| **Auto-eficácia volitiva (de manutenção)** | 1. Estou confiante que amanhã serei capaz de praticar atividade física, mesmo que surjam dificuldades   1 2 3 4 5 6  Discordo totalmente O O O O O O Concordo totalmente |
| **Suporte social** | 1. Tenho quem me apoie a cumprir a atividade física prevista para amanhã   1 2 3 4 5 6  Discordo totalmente O O O O O O Concordo totalmente |
| **Controlo da ação**  **(consciência dos padrões)** | 1. HOJE tive presente o objetivo a que me propus de praticar atividade física e contribuir para os 150 minutos semanais   1 2 3 4 5 6  Discordo totalmente O O O O O O Concordo totalmente |
| **Controlo da ação**  **(adequação do esforço)** | 1. HOJE fiz um esforço para conseguir ir ao encontro da minha intenção de amanhã praticar atividade física, por forma a contribuir para os 150 minutos semanais   1 2 3 4 5 6  Discordo totalmente O O O O O O Concordo totalmente |
| **Atividade Física moderada** | 1. Referente à atividade física moderada praticada hoje (ex. caminhada), refira os minutos totais   ________________________ |
|  | - 1. Pratiquei a seguinte atividade moderada   ________________________ |
|  | - 1. Usei o pedómetro nesta atividade?   O Sim  O Não |
| **Atividade Física vigorosa** | 1. Referente à atividade física vigorosa praticada hoje (atividade física que a deixou ofegante), refira os minutos totais   ________________________ |
|  | - 1. Pratiquei a seguinte atividade vigorosa |
|  | - 1. Usei o pedómetro nesta atividade?   O Sim  O Não |
| **Número de passos** | 1. O meu pedómetro hoje ao final do dia marcou os seguintes passos   _______________________ |
| **Código** | Insira o seu código pessoal  _______________________ |

| **1.2 – Weekly questionnaire in Portuguese** | |
| --- | --- |
| Este questionário pretende conhecer melhor a forma como se sente face à prática de atividade física diária e a quantidade de atividade física que pratica.  Ocupa cerca de 3 minutos a preencher e **deve ser preenchido ao fim do dia.**  Para as primeiras 11 questões (1 a 8 e A, B, C), utilize a escala de 6 pontos, selecionando a opção que mais se adequa a si de acordo com os seguintes significados: 1 "Discordo totalmente"; 2 "Discordo bastante"; 3 "Discordo um pouco"; 4 "Concordo um pouco"; 5 "Concordo bastante"; 6 "Concordo totalmente"  Nas últimas questões preencha os espaços de resposta de acordo com o que foi a Atividade Física realizada hoje. **Muito obrigada pela colaboração.** | |
| **Constructo** | **Questão** |
|  | De modo a ir ao encontro das recomendações da Organização Mundial de Saúde de fazer pelo menos 150 minutos de atividade física semanal: |
| **Perceção de risco** | 1. Sei que posso vir a ter novos problemas de saúde relacionados com o cancro da mama   1 2 3 4 5 6  Discordo totalmente O O O O O O Concordo totalmente |
| **Expectativas e resultados** | 1. Sinto que ao praticar pelo menos 150 minutos de atividade física por semana posso ter benefício face aos problemas relacionados com o cancro da mama   1 2 3 4 5 6  Discordo totalmente O O O O O O Concordo totalmente |
| **Intenção** | 1. Tenho intenção de praticar atividade física amanhã e contribuir para os 150 minutos semanais   1 2 3 4 5 6  Discordo totalmente O O O O O O Concordo totalmente |
| **Autoeficácia de ação** | 1. Estou confiante que amanhã serei capaz de praticar atividade física   1 2 3 4 5 6  Discordo totalmente O O O O O O Concordo totalmente |
| **Planeamento de ação (action planning)** | 1. Já planeei quando, onde e como conseguir praticar atividade física amanhã   1 2 3 4 5 6  Discordo totalmente O O O O O O Concordo totalmente |
| **Identificação de estratégias (planeamento de coping)** | 1. Tenho planos para ultrapassar as barreiras que amanhã possam surgir e que me impeçam de praticar atividade física   1 2 3 4 5 6  Discordo totalmente O O O O O O Concordo totalmente |
| **Autoeficácia volitiva (de manutenção)** | 1. Estou confiante que amanhã serei capaz de praticar atividade física, mesmo que surjam dificuldades   1 2 3 4 5 6  Discordo totalmente O O O O O O Concordo totalmente |
| **Autoeficácia de Recuperação** | 1. Uma vez que comece a praticar atividade física regular, serei capaz de manter essa prática, mesmo que interrompa por algum tempo   1 2 3 4 5 6  Discordo totalmente O O O O O O Concordo totalmente |
| **Suporte social** | 1. Tenho quem me apoie a cumprir a atividade física prevista para amanhã   1 2 3 4 5 6  Discordo totalmente O O O O O O Concordo totalmente |
| **Controlo da ação**  **(consciência dos padrões)** | 1. HOJE tive presente o objetivo a que me propus de praticar atividade física e contribuir para os 150 minutos semanais   1 2 3 4 5 6  Discordo totalmente O O O O O O Concordo totalmente |
| **Controlo da ação**  **(adequação do esforço)** | 1. HOJE fiz um esforço para conseguir ir ao encontro da minha intenção de amanhã praticar atividade física, por forma a contribuir para os 150 minutos semanais   1 2 3 4 5 6  Discordo totalmente O O O O O O Concordo totalmente |
| **Atividade Física moderada** | 1. Referente à atividade física moderada praticada hoje (ex. caminhada), refira os minutos totais   ________________________ |
|  | - 1. Pratiquei a seguinte atividade moderada   ________________________ |
|  | - 1. Usei o pedómetro nesta atividade?   O Sim  O Não |
| **Atividade Física vigorosa** | 1. Referente à atividade física vigorosa praticada hoje (atividade física que a deixou ofegante), refira os minutos totais   ________________________ |
|  | - 1. Pratiquei a seguinte atividade vigorosa |
|  | - 1. Usei o pedómetro nesta atividade?   O Sim  O Não |
| **Número de passos** | 1. O meu pedómetro hoje ao final do dia marcou os seguintes passos   _______________________ |
| **Código** | Insira o seu código pessoal  _______________________ |

| **1.3 – Daily questionnaire in English** | |
| --- | --- |
| This questionnaire aims to know better how you feel about the practice of daily physical activity and the amount of physical activity you practice.  It takes about 2 minutes to complete, and **it should be completed at the end of the day.**  For the first 8 questions, use the 6-point scale, selecting the option that best suits you according to the following meanings: 1 "I totally disagree"; 2 "I strongly disagree"; 3 "I disagree a little"; 4 "I somewhat agree"; 5 "I strongly agree"; 6 "I totally agree"  From question 9 on, fill in the answer spaces according to the Physical Activity performed today.  **Thank you very much for your collaboration.** | |
| **Item** | **Question** |
|  | In order to meet the World Health Organization recommendations to do at least 150 minutes of physical activity weekly: |
| **Behavioral Intention** | 1. I intend to practice physical activity tomorrow and contribute to the weekly 150 minutes   1 2 3 4 5 6  I totally disagree O O O O O O I totally agree |
| **Action Self-efficacy** | 1. I’m confident that tomorrow I will be able to practice physical activity   1 2 3 4 5 6  I totally disagree O O O O O O I totally agree |
| **Action Planning** | 1. I've already planned when, where, and how to be able to practice physical activity tomorrow   1 2 3 4 5 6  I totally disagree O O O O O O I totally agree |
| **Coping Planning** | 1. I have plans to overcome the barriers that may arise tomorrow to prevent me from practicing physical activity   1 2 3 4 5 6  I totally disagree O O O O O O I totally agree |
| **Maintenance Self-efficacy** | 1. I’m confident that tomorrow I will be able to practice physical activity, even if difficulties arise   1 2 3 4 5 6  I totally disagree O O O O O O I totally agree |
| **Social Support** | 1. I have someone to support me in carrying out the physical activity scheduled for tomorrow   1 2 3 4 5 6  I totally disagree O O O O O O I totally agree |
| **Action control (awareness of one’s own standards)** | 1. TODAY, I was aware of the goal I set myself to practice physical activity and contribute to the 150 minutes per week   1 2 3 4 5 6  I totally disagree O O O O O O I totally agree |
| **Action control (self-regulatory effort)** | 1. TODAY, I made an effort to meet my intention to practice physical activity, in order to contribute to the weekly 150 minutes   1 2 3 4 5 6  I totally disagree O O O O O O I totally agree |
| **Moderate Physical Activity** | 1. Regarding the moderate physical activity practiced today (e.g., walking), note the total minutes   ________________________ |
|  | - 1. I practiced the following moderate activity (e.g., walking)   ________________________ |
|  | - 1. Did you use the pedometer in this activity?   O Yes  O No |
| **Vigorous Physical Activity** | 1. Regarding vigorous physical activity practiced today (physical activity that left you breathless) note the total minutes   ________________________ |
|  | - 1. I practiced the following vigorous activity   _______________________________ |
|  | - 1. Did you use the pedometer in this activity?   O Yes  O No |
| **Step counts** | 1. Today, my pedometer today marked the following steps, at the end of the day   _______________________ |
| **Code** | Insert your personal code  _______________________ |

| **1.4 – Weekly questionnaire in English** | |
| --- | --- |
| This questionnaire aims to know better how you feel about the practice of daily physical activity and the amount of physical activity you practice.  It takes about 3 minutes to complete, and **it should be completed at the end of the day.**  For the first 11 questions (1 to 8 and A, B, C), use the 6-point scale, selecting the option that best suits you according to the following meanings: 1 "I totally disagree"; 2 "I strongly disagree"; 3 "I disagree a little"; 4 "I somewhat agree"; 5 "I strongly agree"; 6 "I totally agree"  From question 9 on, fill in the answer spaces according to the Physical Activity performed today.  **Thank you very much for your collaboration.** | |
| **Item** | **Question** |
|  | In order to meet the World Health Organization recommendations to do at least 150 minutes of physical activity weekly: |
| **Risk Awareness** | 1. I know I may have new health problems related to breast cancer   1 2 3 4 5 6  I totally disagree O O O O O O I totally agree |
| **Outcome Expectancies** | 1. I feel that by doing at least 150 minutes of physical activity a week I can improve breast cancer related problems   1 2 3 4 5 6  I totally disagree O O O O O O I totally agree |
| **Behavioral Intention** | 1. I intend to practice physical activity tomorrow and contribute to the weekly 150 minutes   1 2 3 4 5 6  I totally disagree O O O O O O I totally agree |
| **Action Self-efficacy** | 1. I’m confident that tomorrow I will be able to practice physical activity   1 2 3 4 5 6  I totally disagree O O O O O O I totally agree |
| **Action Planning** | 1. I've already planned when, where, and how to be able to practice physical activity tomorrow   1 2 3 4 5 6  I totally disagree O O O O O O I totally agree |
| **Coping Planning** | 1. I have plans to overcome the barriers that may arise tomorrow to prevent me from practicing physical activity   1 2 3 4 5 6  I totally disagree O O O O O O I totally agree |
| **Maintenance Self-efficacy** | 1. I’m confident that tomorrow I will be able to practice physical activity, even if difficulties arise   1 2 3 4 5 6  I totally disagree O O O O O O I totally agree |
| **Recovery Self-efficacy** | 1. Once I start practicing regular physical activity, I will be able to maintain this practice, even if I stop for a while   1 2 3 4 5 6  I totally disagree O O O O O O I totally agree |
| **Social Support** | 1. I have someone to support me in carrying out the physical activity scheduled for tomorrow   1 2 3 4 5 6  I totally disagree O O O O O O I totally agree |
| **Action control (awareness of one’s own standards )** | 1. TODAY, I was aware of the goal I set myself to practice physical activity and contribute to the 150 minutes per week   1 2 3 4 5 6  I totally disagree O O O O O O I totally agree |
| **Action control (self-regulatory effort )** | 1. TODAY, I made an effort to meet my intention to practice physical activity, in order to contribute to the weekly 150 minutes   1 2 3 4 5 6  I totally disagree O O O O O O I totally agree |
| **Moderate Physical Activity** | 1. Regarding the moderate physical activity practiced today (e.g., walking), note the total minutes   ________________________ |
|  | - 1. I practiced the following moderate activity (e.g., walking)   ________________________ |
|  | - 1. Did you use the pedometer in this activity?   O Yes  O No |
| **Vigorous Physical Activity** | 1. Regarding vigorous physical activity practiced today (physical activity that left you breathless) note the total minutes   ________________________ |
|  | - 1. I practiced the following vigorous activity   _______________________________ |
|  | - 1. Did you use the pedometer in this activity?   O Yes  O No |
| **Step count** | 1. Today, my pedometer today marked the following steps, at the end of the day   _______________________ |
| **Code** | Insert your personal code  _______________________ |
