# Supplementary material for: Testing the HAPA model for predicting daily physical activity of women survivors of breast cancer
Source: J Health Psychol. 2025 Jul 20;31(3):1104–19. doi: 10.1177/13591053251347143 (PMC12949744; doi:10.1177/13591053251347143)
Supplement: sj-docx-2-hpq-10.1177_13591053251347143 – Supplemental material for Testing the HAPA model for predicting daily physical activity of women survivors of breast cancer [file sj-docx-2-hpq-10.1177_13591053251347143.docx]

**Supplemental File 2 - Pilot study for grouping daily assessed HAPA constructs**

The purpose of this pilot study was to examine the extent to which the items we used to measure the HAPA constructs overlapped. This measurement problem has also been the subject of theoretical considerations in previous studies using the HAPA model (Godinho et al., 2014; Hagger and Luszczynska, 2014; Schwarzer and Luszczynska, 2015). Indeed, the variables in the present study are highly correlated, as can be seen from the correlation matrix in Table S2.

| *Table S2 - Correlations of the daily assessed variables* | | | | | | | | |
| --- | --- | --- | --- | --- | --- | --- | --- | --- |
|  | 1. | 2. | 3. | 4. | 5. | 6. | 7. | 8 |
| 1. Intention | - |  |  |  |  |  |  |  |
| 2. Action self-efficacy | ,885^**^ | - |  |  |  |  |  |  |
| 3. Action planning | ,875^**^ | ,892^**^ | - |  |  |  |  |  |
| 4. Coping planning | ,862^**^ | ,891^**^ | ,949^**^ | - |  |  |  |  |
| 5. Maintenance self-efficacy | ,853^**^ | ,882^**^ | ,930^**^ | ,957^**^ | - |  |  |  |
| 6. Social Support | ,521^**^ | ,495^**^ | ,537^**^ | ,537^**^ | ,545^**^ | - |  |  |
| 7. Action control - awareness of the standards | ,663^**^ | ,637^**^ | ,654^**^ | ,637^**^ | ,625^**^ | ,309^**^ | - |  |
| 8. Action control - effort adequacy | ,642^**^ | ,632^**^ | ,628^**^ | ,616^**^ | ,595^**^ | ,328^**^ | ,916^**^ | - |
| *Note*. ***p*< 0.01 | | | | | | | | |

First, the HAPA model includes several constructs, but some of them appear theoretically mergeable. Schwarzer and Luszczynska (2015) mention volitional self-efficacy that grouped both maintenance and recovery self-efficacy, noting that there is a functional difference between these self-efficacy constructs, while their temporal sequence is less important. Moreover, different phase-specific self-efficacy beliefs may be present at the same point in time. We have noted the same between action and maintenance self-efficacy. The same authors refer to action planning and coping planning as different constructs. However, they consider both as a prospective strategy compared to action control, which is considered a concurrent self-regulatory strategy, and three different aspects can be considered in the course of self-regulation: Self-observation, awareness of standards, and adequacy of self-regulation. Hagger and Luszczynska (2014)) also consider both action planning and coping planning as components that serve as mechanisms for implementing intentions.

Second, although the constructs can be individualized, HAPA is not a static model. It is flexible enough as an open architecture, such as we can find the aggregated constructs in several studies. Perceived self-efficacy is considered as a whole in, e.g., dietary behaviors by Gutiérrez-Doña et al. (2009) and fruits and vegetables intake by Godinho et al. (2014). Also action and coping planning are often considered together as planning strategies for, e.g., fruits and vegetables consumption by Gholami et al. (2013) and Lange et al. (2013) or for sedentary behaviors by Maher & Conroy, 2016) and physical activity by Parschau et al. (2012).

The current pilot study was undertaken with a different sample of eleven adult women without illiteracy or the presence of cognitive deficits that would make it impossible to understand the purpose of the study. Their ages were between 37 and 59 years old (*M* = 45, *SD* =7.2). Nine had graduated from university and two had completed secondary school. The sample was recruited by convenience, and they agreed to participate. An individual meeting was set, and the investigator presented the aims of the study, its potential benefits, the rights of the participants, and consent to participate. Sociodemographic data were collected (age and level of education).

Each of the items of the daily-assessed constructs was written on a separate card; participants were asked to organize them, following the instruction: “After carefully reading the eight sentences of the eight cards presented, please organize them according to the idea in common that they convey to you. You can do this between one and eight sets: only 1 set in case all sentences suggest the same idea to you (that is, if you think all sentences ask the same thing); 2, 3, 4, 5, 6, or 7 sets depending on the number of ideas that the sentences convey to you; 8 sets if each sentence conveys a different idea to you (that is, if you think all the sentences ask about different issues)”.

Multidimensional Scaling (MDS) and Cluster Analysis (CA) (SPSS version 25) were used with the stimuli provided (one item per card). After the association of constructs, the following variables were found: intention, self-efficacy (including action self-efficacy and maintenance self-efficacy, planning (including action and coping planning), action control (both items), and social support. The results shown in Table S2 confirm this aggregation, as the strong positive correlations between the aggregated variables show that they strongly overlap therefore they should not be included separately in the models, because they cause multicollinearity.

References:

Gholami, M., Lange, D., Luszczynska, A., Knoll, N., & Schwarzer, R. (2013). A dietary planning intervention increases fruit consumption in Iranian women. *Appetite*, *63*, 1–6. https://doi.org/10.1016/j.appet.2012.12.005

Godinho, C. A., Alvarez, M.-J., Lima, M. L., & Schwarzer, R. (2014). Will is not enough: coping planning and action control as mediators in the prediction of fruit and vegetable intake. *British Journal of Health Psychology*, *19*(4), 856–870. https://doi.org/10.1111/bjhp.12084

Gutiérrez-Doña, B., Lippke, S., Renner, B., Kwon, S., & Schwarzer, R. (2009). Self-Efficacy and Planning Predict Dietary Behaviors in Costa Rican and South Korean Women: Two Moderated Mediation Analyses. *Applied Psychology: Health and Well-Being*, *1*(1), 91–104. https://doi.org/10.1111/j.1758-0854.2008.01001.x

Hagger, M. S., & Luszczynska, A. (2014). Implementation Intention and Action Planning Interventions in Health Contexts: State of the Research and Proposals for the Way Forward. *Applied Psychology: Health and Well-Being*, *6*(1), 1–47. https://doi.org/10.1111/aphw.12017

Lange, D., Richert, J., Koring, M., Knoll, N., Schwarzer, R., & Lippke, S. (2013). Self-regulation prompts can increase fruit consumption: A one-hour randomised controlled online trial. *Psychology and Health*, *28*(5), 533–545. https://doi.org/10.1080/08870446.2012.751107

Maher, J. P., & Conroy, D. E. (2016). A dual-process model of older adults’ sedentary behavior. *Health Psychology*, *35*(3), 262–272. https://doi.org/10.1037/hea0000300

Parschau, L., Richert, J., Koring, M., Ernsting, A., Lippke, S., & Schwarzer, R. (2012). Changes in social-cognitive variables are associated with stage transitions in physical activity. *Health Education Research*, *27*(1), 129–140. https://doi.org/10.1093/her/cyr085

Schwarzer, R., & Luszczynska, A. (2015). Health Action Process Approach. In M. Conner & P. Norman (Eds.), *Predicting and changing health behaviour-Research and Practice with Social Cognition Models* (3rd ed.). McGraw Hill Education - Open University Press.
